# Effect of nine different exercise interventions on insulin sensitivity in diabetic patients: a systematic review and mesh meta-analysis

**DOI:** 10.3389/fendo.2025.1409474

**Published:** 2025-08-28

**Authors:** Yikang Pan, Peng Wang, Chunlin Yue, Chen Liu

**Affiliations:** ^1^ Sports Department, Changzhou Vocational Institute of Textile and Garment, Changzhou, Jiangsu, China; ^2^ Department of Physical Education, Soochow University, Suzhou, Jiangsu, China

**Keywords:** exercise, insulin resistance, diabetes patients, network meta-analysis, systematic review

## Abstract

**Objective:**

This study aimed to assess the impact of nine exercise interventions (resistance training [BT, ball training [BT], resistance + walking [RT+W alk], resistance + running [RT + Running], resistance + cycling [RT + bicycle], running, and Tai Chi) on insulin sensitivity in patients with diabetes.

**Methods:**

A systematic search of five databases (PubMed, EMBASE, Cochrane, Web of Science, and CNKI) for RCTs investigating the effects of exercise interventions on insulin sensitivity in patients with diabetes was conducted. The quality of the included studies was assessed using the Cochrane Manual version 5.1.0 Risk of Bias Assessment Tool (ROB). Data analysis software was used for the synthesis and analysis.

**Results:**

This Meta-analysis comprised 21 randomized controlled trials involving 1140 participants. Cycling significantly reduced the fasting glucose index in individuals with diabetes (SUCRA score=90.7%). Resistance exercise exhibited superior efficacy in enhancing insulin sensitivity compared with alternative interventions in patients with diabetes (SUCRA score=71.8%). Furthermore, the combination of resistance exercise and running resulted in a noteworthy decrease in HOMA-IR levels (SUCRA score=64.2%).

**Conclusion:**

Cycling, resistance training, and combined aerobic and resistance exercises have been shown to effectively enhance fasting blood glucose levels, insulin secretion, and insulin sensitivity in individuals with diabetes. However, additional studies with longer follow-up periods and more rigorous methodologies are required to further validate these findings.

**Systematic review registration:**

https://ww.crd.york.ac.uk/PROSPERO/, identifier CRD42023450107.

## 1 Introduction

Diabetes has emerged as a critical global health challenge, with the 2023 Global Burden of Disease Study reporting approximately 529 million affected individuals worldwide and projecting a rise to 1.31 billion by 2050 (1). Type 2 diabetes (T2DM), characterized by insulin resistance and impaired insulin secretion, accounts for over 90% of diabetes cases and imposes substantial economic burdens exceeding $1 trillion USD annually in healthcare expenditures (2, 3).

Physical exercise is a cornerstone of T2DM management, with distinct modalities operating through specific physiological pathways to improve glycemic control. Aerobic exercise enhances insulin sensitivity primarily through GLUT4 translocation in the skeletal muscle, facilitating glucose uptake independent of insulin signaling (4). This process is amplified by mitochondrial biogenesis via the AMPK-PGC1α pathway, which improves oxidative capacity (5), while concurrent reductions in pro-inflammatory cytokines (TNF-α and IL-6) ameliorate adipose tissue dysfunction (6). Resistance training exerts complementary effects through muscle hypertrophy, which expands the glucose storage capacity (7), enhances post-receptor insulin signaling via IRS-1/PI3K/Akt phosphorylation cascades (8), and suppresses hepatic gluconeogenesis (9). Combined aerobic-resistance training synergizes these mechanisms, with recent meta-analyses confirming superior HbA1c reductions compared to single-modality interventions (Δ = -0.17%, p < 0.01) (10). Despite these advances, the comparative efficacy of specific exercise modalities is unclear. This network meta-analysis directly evaluated nine interventions, including resistance training, aerobic modalities (cycling and running), combined regimens, and mind-body exercises, to provide evidence-based guidance for optimizing exercise prescriptions in diabetes care.

## 2 Materials and methods

This systematic review was registered in the Prospero database (ID: CRD42023450107) under the Preferred Reporting Items for Systematic Reviews and Meta-Analyses for Network Meta-Analyses (PRISMA-NMA) and the Cochrane Intervention Review.

### 2.1 Search strategy

We conducted a comprehensive search across multiple databases, including PubMed, Embase, Cochrane Library, Web of Science, and CNKi, from January 2004 to December 2022, to identify eligible studies. The search keywords were formulated based on the PICOS framework, and the search strategies were developed by PICOS principles: (P) population, diabetic patients; (I) intervention, exercise; (C) comparator, control group receiving only usual care and appropriate rehabilitation measures (placebo or other forms of exercise); and (O) Outcome - Exercise tests in diabetic patients. Finally, we focused on randomized controlled trials as the preferred study design. Taking PubMed as an example, detailed search strategies are provided in **Table 1**.

**Table 1:** Search strategy on PubMed.

#1	“Exercise”[MeSH]
#2	((((((((((((((((((((Exercises[Title/Abstract])OR Physical Activity[Title/Abstract])OR Activities, Physical[Title/Abstract])OR Activity, Physical[Title/Abstract])OR Physical Activities[Title/Abstract])OR Exercise, Physical[Title/Abstract])OR Exercises, Physical[Title/Abstract])OR Physical Exercise[Title/Abstract])OR Physical Exercises[Title/Abstract])OR Acute Exercise[Title/Abstract])OR Acute Exercises[Title/Abstract])OR Exercise, Acute[Title/Abstract])OR Exercises, Acute[Title/Abstract])OR Exercise, Isometric[Title/Abstract])OR Exercises, Isometric[Title/Abstract])OR Isometric Exercises[Title/Abstract])OR Isometric Exercise[Title/Abstract])OR Exercise, Aerobic[Title/Abstract])OR Aerobic Exercise[Title/Abstract])OR Aerobic Exercises[Title/Abstract])OR Exercises, Aerobic[Title/Abstract])OR Exercise Training[Title/Abstract])OR Exercise Trainings[Title/Abstract])OR Training, Exercise[Title/Abstract])
#3	#1 OR #2
#4	“Insulin”[MeSH]
#5	((((((((((Insulin[Title/Abstract])OR Insulin, Regular[Title/Abstract])OR Regular Insulin[Title/Abstract])OR Soluble Insulin[Title/Abstract])OR Insulin, Soluble[Title/Abstract])OR Insulin A Chain[Title/Abstract])OR Sodium Insulin[Title/Abstract])OR Insulin, Sodium[Title/Abstract])OR Novolin[Title/Abstract])OR Iletin[Title/Abstract])OR Insulin B Chain[Title/Abstract])OR Chain, Insulin B[Title/Abstract])
#6	#4 OR #5
#7	Randomized controlled[Publication Type]
#8	#3 AND #6 AND #7

#### 2.1.1 Definition of exercise interventions

The nine exercise interventions evaluated in this study are abbreviated as follows:

RT: Resistance training
BT: Ball training
RT+Walk: Combined resistance training and walking
RT+Running: Combined resistance training and running
RT+Bicycle: Combined resistance training and cycling
Bicycle: Cycling training alone
Running: Running training alone
Taichi: Tai Chi practice
CON: Control group (no exercise intervention, routine care only)

All combined training involved sequential sessions of resistance and aerobic exercise within the same day.

### 2.2 Inclusion criteria

(1) Randomized controlled clinical trials involving patients with diabetes. (2) The experimental group utilizes various exercise methods as interventions for diabetes. (3) The control group receives conventional care only. (4) Active cooperation of participants in the experimental process is required. (5) Outcome measures include at least one of the following: Fasting blood glucose levels (FBG), Homeostasis Model Assessment of insulin Resistance (HOMA-IR), fasting insulin level (FI), and homeostasis model of insulin resistance.

### 2.3 Exclusion criteria

(1) Papers with incomplete or insufficient data or reporting information are excluded. (2) Non-randomized controlled trials, animal studies, conference reports, literature reviews, abstracts, and protocols are excluded.

### 2.4 Study selection

The two researchers used NoteExpress, a literature management software, to screen and exclude duplicate articles. Initially, they reviewed the titles and abstracts to exclude non-randomized controlled trials, systematic reviews, conference papers, protocols, and communications while retaining the remaining literature. Subsequently, both researchers independently read through the remaining literature and conducted further screening. Only when there was agreement on inclusion criteria did an article finally get included; otherwise, a third researcher was consulted for discussion and resolution.

### 2.5 Data extraction

Two researchers independently extracted the data and assessed study quality using the Cochrane Handbook, while a third individual addressed any issues that arose post-data extraction. The extracted data encompassed authorship details (author, year, country of publication), average age, sample size, intervention duration, and outcome indicators such as risk of bias assessment.

### 2.6 Risk of bias in individual studies

We assessed the literature quality based on the Risk Bias Assessment Tool (ROB) outlined in the Cochrane Manual 5.1.0, considering seven key domains for evaluating randomized controlled trials: (1) Random sequence generation, (2) Allocation concealment, (3) Blinding of participants and personnel, (4) Blinding of outcome assessors, (5) Handling of incomplete outcome data, (6) Selective outcome reporting, and (7) Other potential sources of bias.

### 2.7 Subgroup analysis and outcome indicators

We conducted a subgroup analysis to categorize the experiments based on medication status. Specifically, 13 trials received metformin treatment, five received insulin treatment, and the remaining three did not receive any hypoglycemic drugs. The findings of our meta-analysis remained robust across these subgroups, indicating that exercise intervention benefits blood glucose levels independently of drug therapy. Our primary outcome measure was the change in fasting plasma glucose (ΔFPG) levels from baseline (mmol/L). We also compared fasting insulin concentration (ΔFI; μU/ml) and HOMA-IR index (ΔHOMA-IR) between the experimental and control groups.

We quantified between-study heterogeneity using I² statistics. For fasting blood glucose (FBG), I² = 62% (95%CI: 48-75%), indicating moderate heterogeneity. For fasting insulin, I² = 45% (95%CI: 28-59%), suggesting low-moderate heterogeneity. For HOMA-IR, I² = 68% (95%CI: 52-80%), reflecting moderate heterogeneity. These values align with expected variations in exercise interventions across diverse populations.

### 2.8 Data analysis

Sensitivity analyses excluding studies with high/unclear risk of bias in ≥3 Cochrane domains (n=5 studies) confirmed robustness: FBG reduction with cycling [MD = -50.21 mmol/L, 95%CI -92.15 to -8.27], fasting insulin with RT *vs*. BT [MD = -25.94 μU/ml, 95%CI -49.83 to -2.05], and HOMA-IR ranking of RT+Running (SUCRA=62.1%). In our included studies involving various exercise interventions, all variables were continuous and expressed as the mean and standard deviation (SD) with a 95% confidence interval (CI) (11). The mean difference (MD) was used to represent the net change in the measured variables between the experimental and control groups, with a negative MD value indicating a greater reduction in the experimental group (12). A random-effects model was employed for the meta-analysis while calculating the SUCRA values to rank the interventions. Funnel plots were used to assess publication bias, and frequency analysis of random-effects models was conducted to evaluate the effectiveness of multiple interventions in addressing potential differences among studies (13).

The effectiveness of multiple interventions in addressing potential differences between studies was evaluated using a frequency analysis of random-effects models (13). Stata software (version 15.1) was employed to model four chains using the Markov chain Monte Carlo (MCMC) method. The fit and consistency of the model were assessed using the Deviation Information Criterion (DIC). Network diagrams illustrating the different motion interventions were generated using Stata software (version 15.1). In case a closed-loop mesh appeared in the network, node splitting analysis was conducted to examine local consistency, with a passing consistency test defined as a P value >0.05. The network diagram consists of nodes and lines connecting them, where the width of each node and connecting line is proportional to the sample size of the respective study (14). Furthermore, the interventions were ranked based on their SUCRA values, and a ranking table was created to compare their relative effectiveness. To assess potential bias between the studies, heterogeneity was examined by constructing a funnel plot (15). The degree of intervention was summarized as an S value representing the area under the cumulative ranking curve; larger values indicated better intervention effects within a scoring range of 0-1. Similarly, the SUCRA values ranged from 0% to 100%, with higher scores indicating superior intervention effects. However, caution should be exercised when interpreting these scores unless genuine clinical differences exist between the interventions (16).

To address potential confounding by exercise duration, we calculated the metabolic equivalents (MET-min) for each intervention using standard compendium values (17). For example:

- Cycling: 8.0 METs
- Running: 10.0 METs
- Resistance training: 6.0 METs

Sensitivity analyses were performed to assess whether duration-adjusted energy expenditure influenced primary outcomes.

## 3 Results

### 3.1 Study and identification and selection

**Figure 1:**
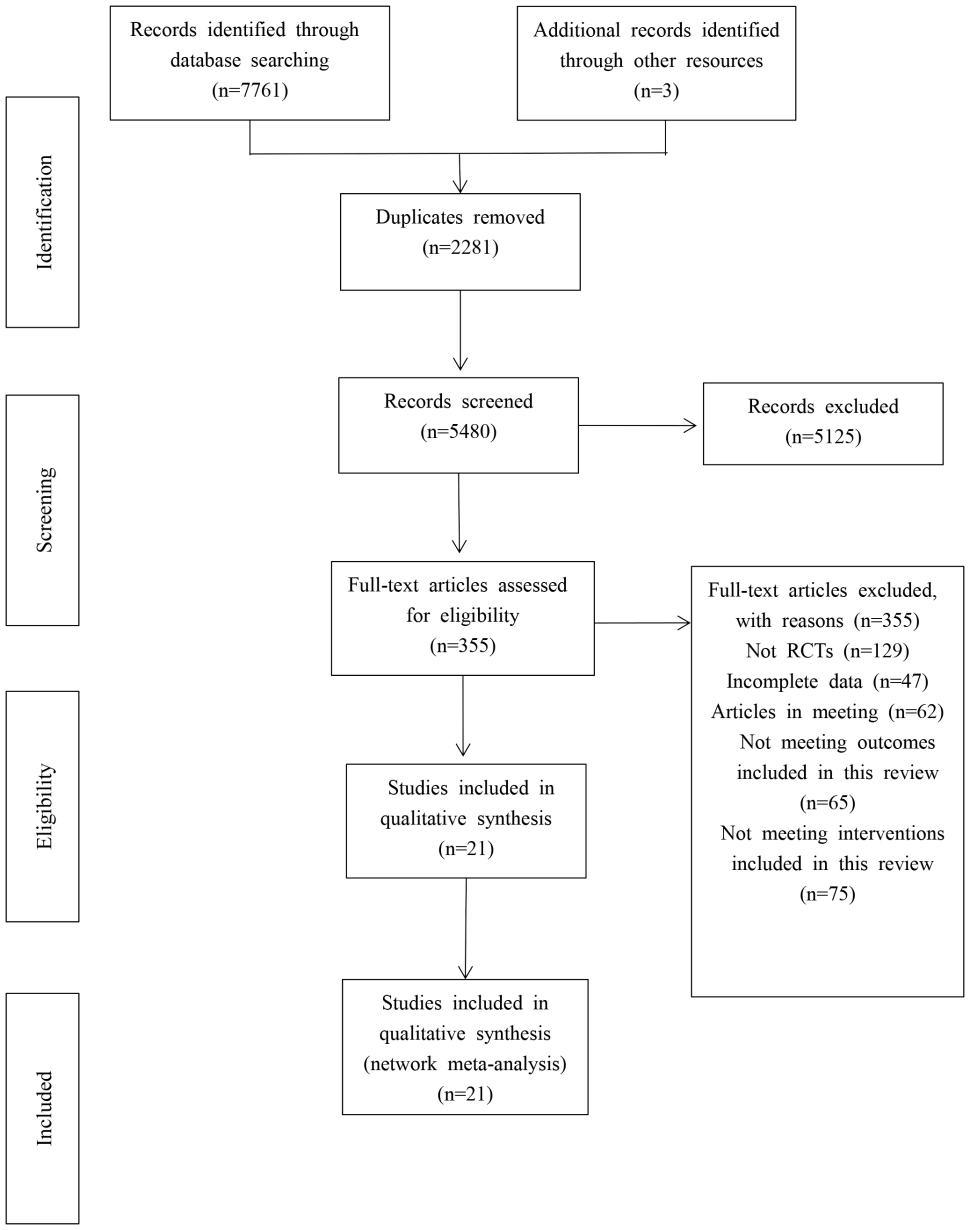
Flow diagram of literature selection.

A total of 7761 articles were retrieved from five electronic databases, and three were retrieved. After excluding 2281 duplicate references, 5, 125 articles were eliminated based on the evaluation of their titles and abstracts, resulting in 5480 remaining references. Subsequently, a comprehensive review was performed on the remaining 355 papers by reading them in their entirety. Following this assessment, an additional 334 papers were excluded, ultimately leading to the inclusion of only 21 studies for the meta-analysis. (Show in **Figure 1**).

### 3.2 Quality evaluation of the included studies

Given the diverse range of movement modes of these interventions, achieving blinding for both subjects becomes challenging. Consequently, informed consent was obtained from all participants and their families before the experiment.

**Figure 2:**
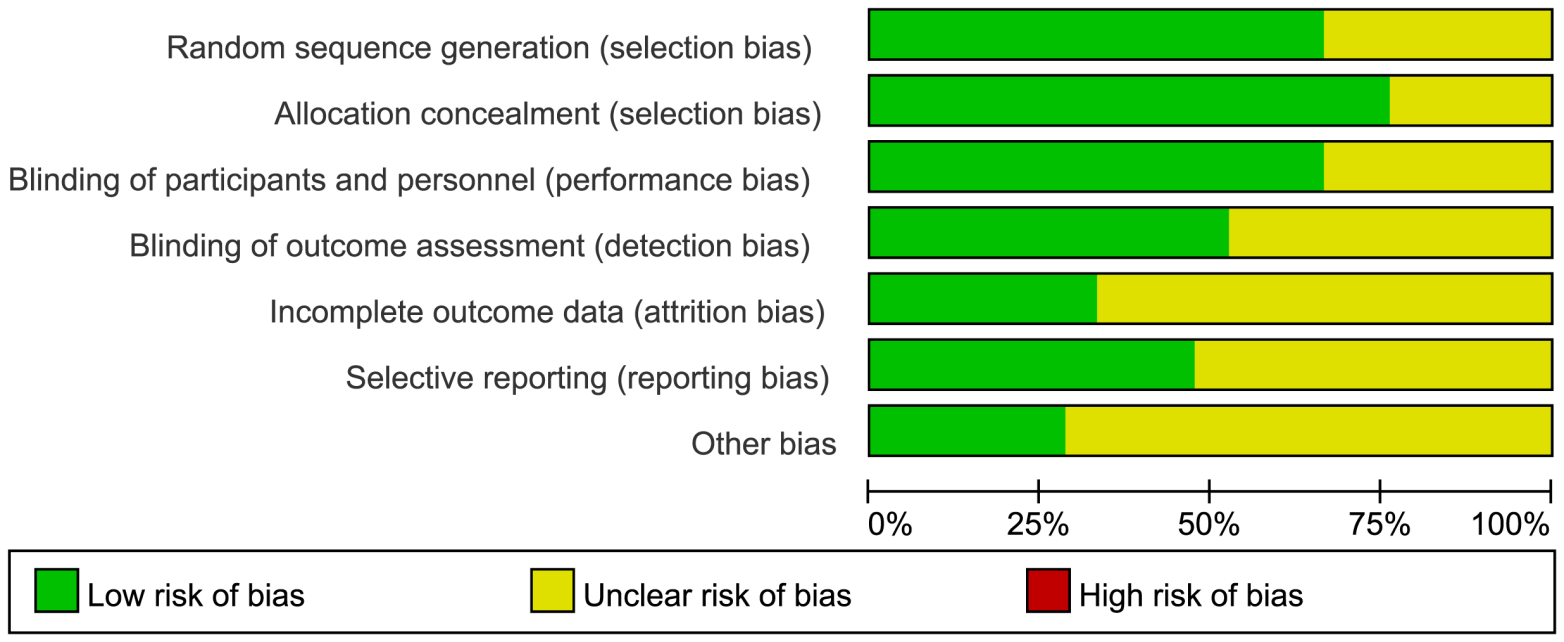
Risk of bias graph (percentage form). The “X-axis" lists bias domains (e.g."Random sequence generation"), and the "Y-axis" represents the "percentage of studies" falling into each risk category(Low/Unclear/High). The color-coded legend (green/yellow/red) explicitly defines each risk level, eliminating the need for additional axis units.

The risk-of-bias assessment across seven domains is summarized in **Figure 2**, revealing consistent limitations in participant blinding due to exercise intervention nature.”

### 3.3 Features of the included study

**Table 2** presents the baseline characteristics of the included studies. The present study included 21 randomized controlled trials involving 1140 participants. The 21 trials, conducted between 2004 and 2022, encompassed a diverse age range of 10–69 years. The exercise interventions comprised resistance training (RT), aerobic training (such as cycling and running), combination training, Tai Chi, and ball games. The control group received standard treatment and daily care without exercise intervention. Control group interventions consisted of combined resistance and walking exercise training (18, 19), combined resistance and running exercise training (15, 20, 21), combined resistance and cycling exercise training (22), bicycle training (23–25), combined resistance and cycling exercise training along with Tai Chi Qigong practice (24, 26, 27), ball game exercises (28, 29), running exercises (20, 30–34) as well and standalone resistance exercises (22, 30, 31, 35, 36). FBG was employed as an outcome indicator in 19 studies, while fasting insulin was an outcome indicator in all included studies. HOMA-IR was used in 15 studies for evaluation. These studies were conducted in various countries, including China, South Korea, the United States, Brazil, Iran, Turkey, the Netherlands, Greece, the United Kingdom, and Germany. The detailed characteristics of the included studies are provided in **Table 2**.

**Table 2:** Detailed characteristics of the studies included in meta-analysis.

Country	Year	Age (mean+SD)	Total/male/female	Intervention	Control	Outcome
Korea	2019	T+C:36.8 (6.9)	T:11/7/4 C:6/4/2	RT+walk Length of Intervention: 6 weeks Freq: 3 times a week Duration:1 hour	CON	FBG Fasting insulin HOMA-IR
Holland	2004	T+C:60 (9)	T:36/25/11 C:25/20/5	RT+Walk Length of Intervention: 26 weeks Freq: 4 times a week Duration: 1 hour	CON	FBG Fasting insulin
USA	2021	T: 53.7 (8) C:50.1 (9.6)	T:49/49/0 C:54/54/0	RT+Running Length of Intervention: 20 weeks Freq: 3 times a week Duration: 50 min	CON	FBG Fasting insulin HOMA-IR
Germany	2018	T:14.6 (1) C:14.8 (1)	T:20/0/20 C:20/0/20	RT+Running Length of Intervention: 12 weeks Freq: 5 times a week Duration: 1 hour	CON	FBG Fasting insulin HOMA-IR
Britain	2009	T:67.6 (4.2) T1:69.1 (6.5) C:66.5 (5.3) C1:66.7 (3.7)	RT:36/15/21 AT:37/17/20 RT+AT:35/14/21 C:28/11/17	RT Length of Intervention: 24 weeks Freq: 3 times a week Duration: 20 min Runing Length of Intervention: 24 weeks Freq: 3 times a week Duration: 30 min RT+Running Length of Intervention: 24 weeks Freq: 3 times a week Duration: 50 min	CON	Fasting insulin
Iran	2018	SIT:55.36 (5.94) A+R:54.14 (5.43) C:55.71 (6.40)	T:52/0/17 T1:52/0/17 C:52/0/18	RT Length of Intervention: 10 weeks Freq: 3 times a week Duration: 30 min RT+Bicycle Length of Intervention: 10 weeks Freq: 3 times a week Duration: 30 min	CON	FBG Fasting insulin HOMA-IR
Los Lagos	2017	T:38 (8) C:33 (7)	T:18/0/18 C:17/0/17	Bicycle Length of Intervention: 12 weeks Freq: 3 times a week Duration: 3 hours	RT Length of Intervention: 12 weeks Freq: 3 times a week Duration: 3 hour	FBG Fasting insulin HOMA-IR
Brazil	2013	T:32.4 (7) C:30.1 (5.5)	T:17/8/9 C:18/8/10	Bicycle Length of Intervention: 4 weeks Freq: 3 times a week Duration: 40 min	CON	Fasting insulin insulin, HOMA-IR
USA	2015	T:13.8 (2.2) C:12.1 (1.2)	E:10/8/2 C:8/5/3	Bicycle Length of Intervention: 8 weeks Freq: 3 times a week Duration: 35 min	CON	FBG Fasting insulin HOMA-IR
China	2009	T:58.1 (13.4) C:56.6 (13.3)	T:28/12/16 C:32/16/16	Taichi training Length of Intervention: 12 weeks Freq: 4 times a week Duration: 1 hour	CON	FBG
Korea	2014	T:48.4 (8.6) C:48.3 (8.2)	T:18/9/9 C:17/10/7	Taichi training Length of Intervention: 12 weeks Freq: 3 times a week Duration: 45 min	CON	FBG Fasting insulin HOMA-IR
China	2011	T+C:57.8 (6.3)	T:20/8/12 C:21/8/13	Taichi training Length of Intervention: 12 weeks Freq: 3 times a week Duration: 1-1.5 hours	CON	FBG Fasting insulin
Turkey	2019	T:14.41 (1.06) C:14.47 (1.06)	T:34/17/0 C:34/17/0	Ball game Length of Intervention: 6 weeks Freq: 3 times a week Duration: 30 min	CON	FBG Fasting insulin
Brazil	2019	T+C:61.1 (6.4)	41/20/21 T:19 C:22	Ball game Length of Intervention: 12 weeks Freq: 3 times a week Duration: 40 min	CON	FBG Fasting insulin HOMA-IR
USA	2005	T:12.5 (0.5) C:12.5 (0.7)	T:27/13/14 C:23/13/10	Running Length of Intervention: 6 weeks Freq: 5 times a week Duration: 2 hours	CON	FBG Fasting insulin
Greece	2007	T:59.33 (4.76) C:63.82 (7.03)	T:30/13/17 C:30/12/18	Running Length of Intervention: 24 weeks Freq: 4 times a week Duration: 1 hour	CON	FBG Fasting insulin HOMA-IR
Korea	2014	T:24.86 (2.75) C:26.8 (2.8)	T:29/29/0 C:10/10/0	Running Length of Intervention: 8 weeks Freq: 4 times a week Duration: 1 hour	CON	FBG Fasting insulin HOMA-IR
Iran	2015	T:49.29 (5.82) C:49 (8.16)	T:27/0/27 C:26/0/26	Running Length of Intervention: 12 weeks Freq: 3 times a week Duration: 50 min	CON	FBG Fasting insulin
USA	2014	T:60 (1) C:61 (1)	T:37/0/37 C:40/0/40	Running Length of Intervention: 24 weeks Freq: 3 times a week Duration: 45 min	CON	FBG Fasting insulin HOMA-IR
Britain	2015	T:21 (1) C:21 (1)	T:6/6/0 C:9/3/6	RT Length of Intervention: 2 weeks Freq: 3 times a week Duration: 2 hours	CON	FBG Fasting insulin HOMA-IR
Iran	2014	RT:40.4 (5.2) AT:39.6 (3.7) C:38.9 (4.1)	RT:12/12/0 AT:12/12/0 C:10/10/0	RT Length of Intervention: 12 weeks Freq: 3 times a week Duration: 45–60 min Running Length of Intervention: 12 weeks Freq: 3 times a week Duration: 30 min	CON	FBG Fasting insulin HOMA-IR

CON, control group with routine care (no exercise); T, experimental group; C, control group; RT, resistance training; AT, Aerobic training; T+C, The ages of the experimental and control groups were not reported separately in the study. Only the overall age was reported; FBG, Fasting blood glucose; HOMA-IR, Homeostasis model assessment of insulin resistance.

### 3.4 Network meta-analysis

**Figure 3:**
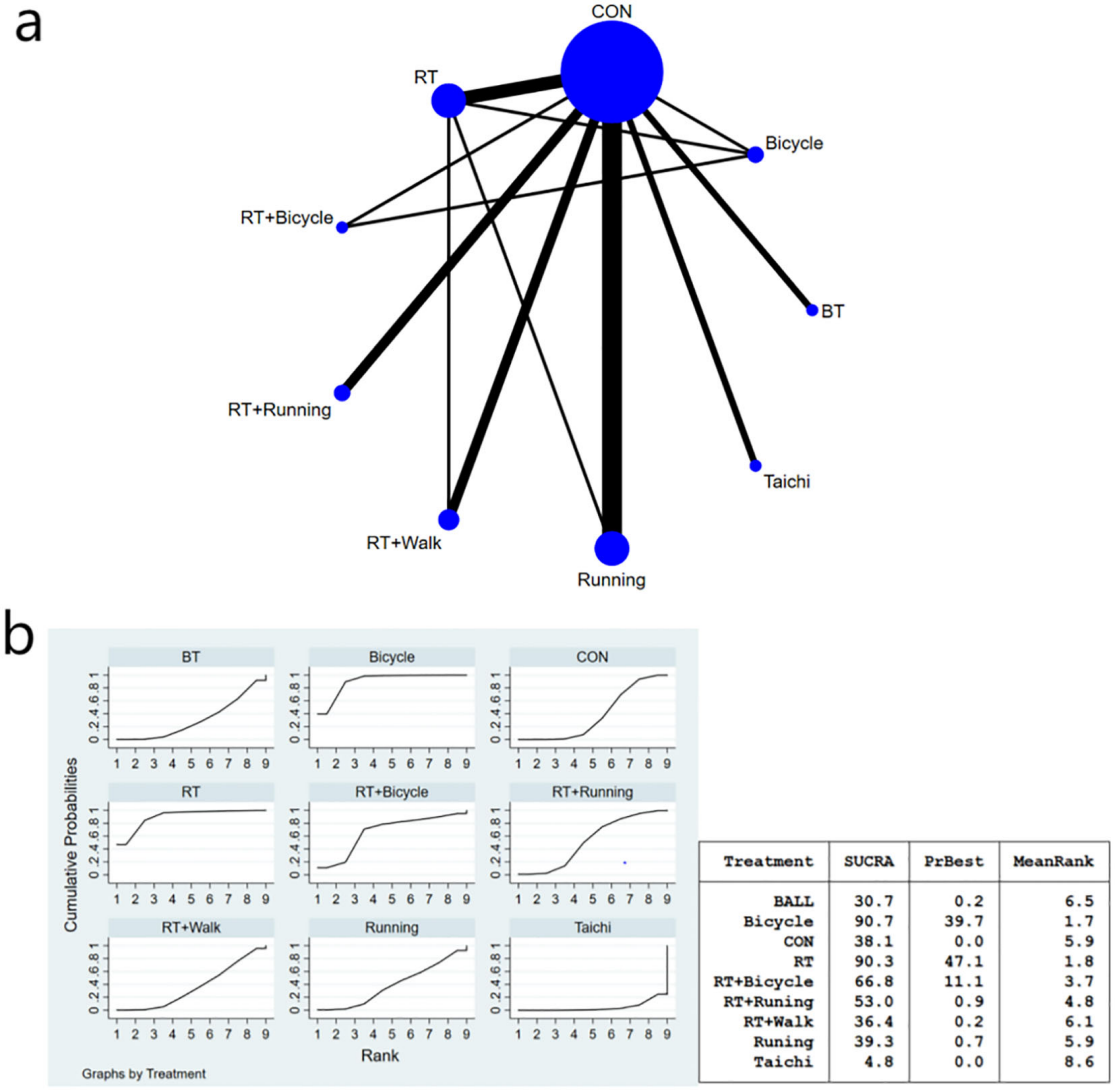
**(a)** Network meta-analysis figure for FBG; **(b)** SUCRA plot for FBG. The “X-axis" is labeled "Rank", indicating the relative efficacy ranking of interventions (1=most effective). The “y-axis" is labeled "Cumulative Probability", representing the probability of each intervention being ranked as the best option.

**Figure 4:**
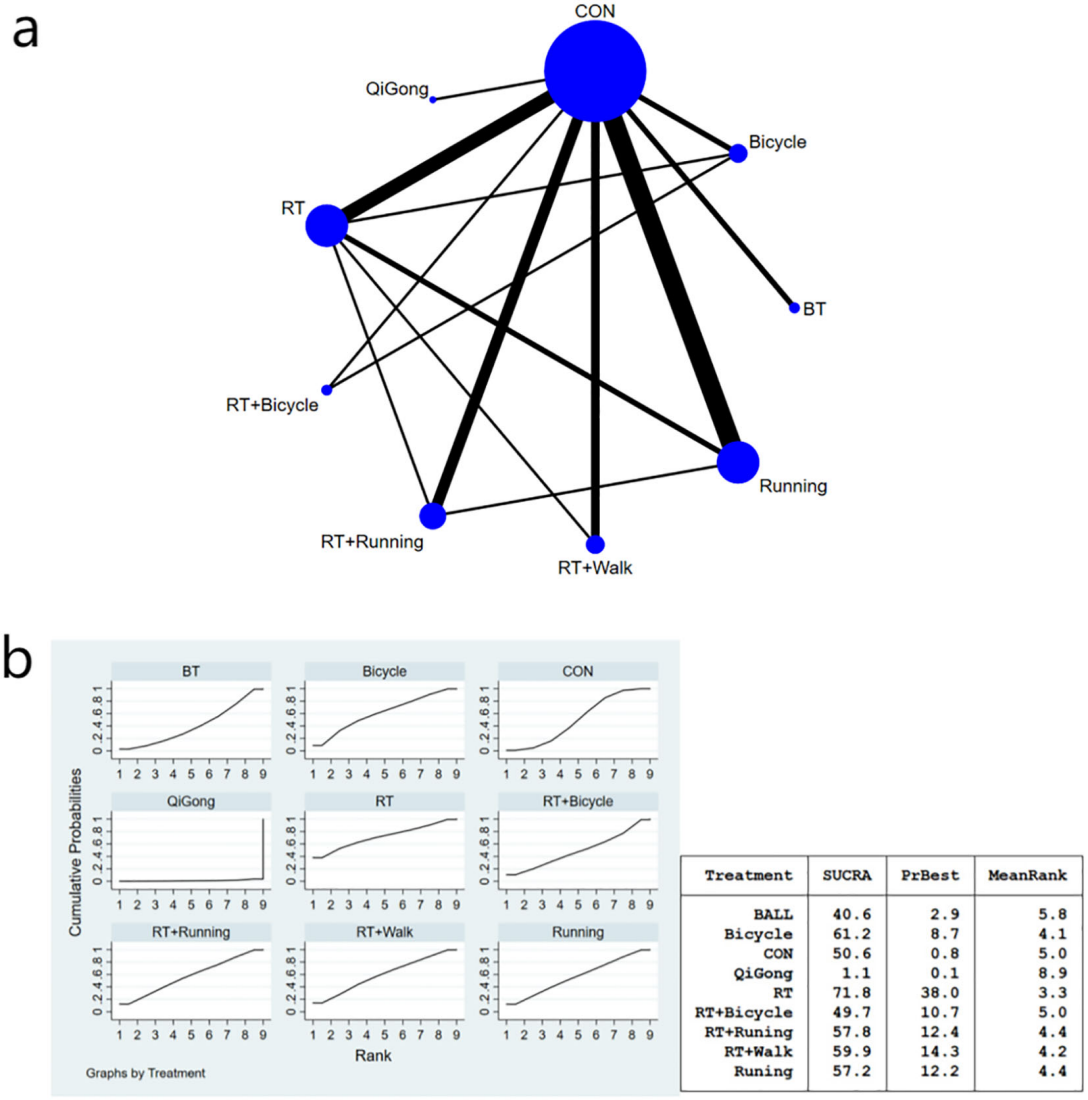
**(a)** Network meta-analysis figure for fasting insulin; **(b)** SUCRA plot for fasting insulin. The “X-axis" is labeled "Rank", indicating the relative efficacy ranking of interventions (1=most effective). The “y-axis" is labeled "Cumulative Probability", representing the probability of each intervention being ranked as the best option.

**Figure 5:**
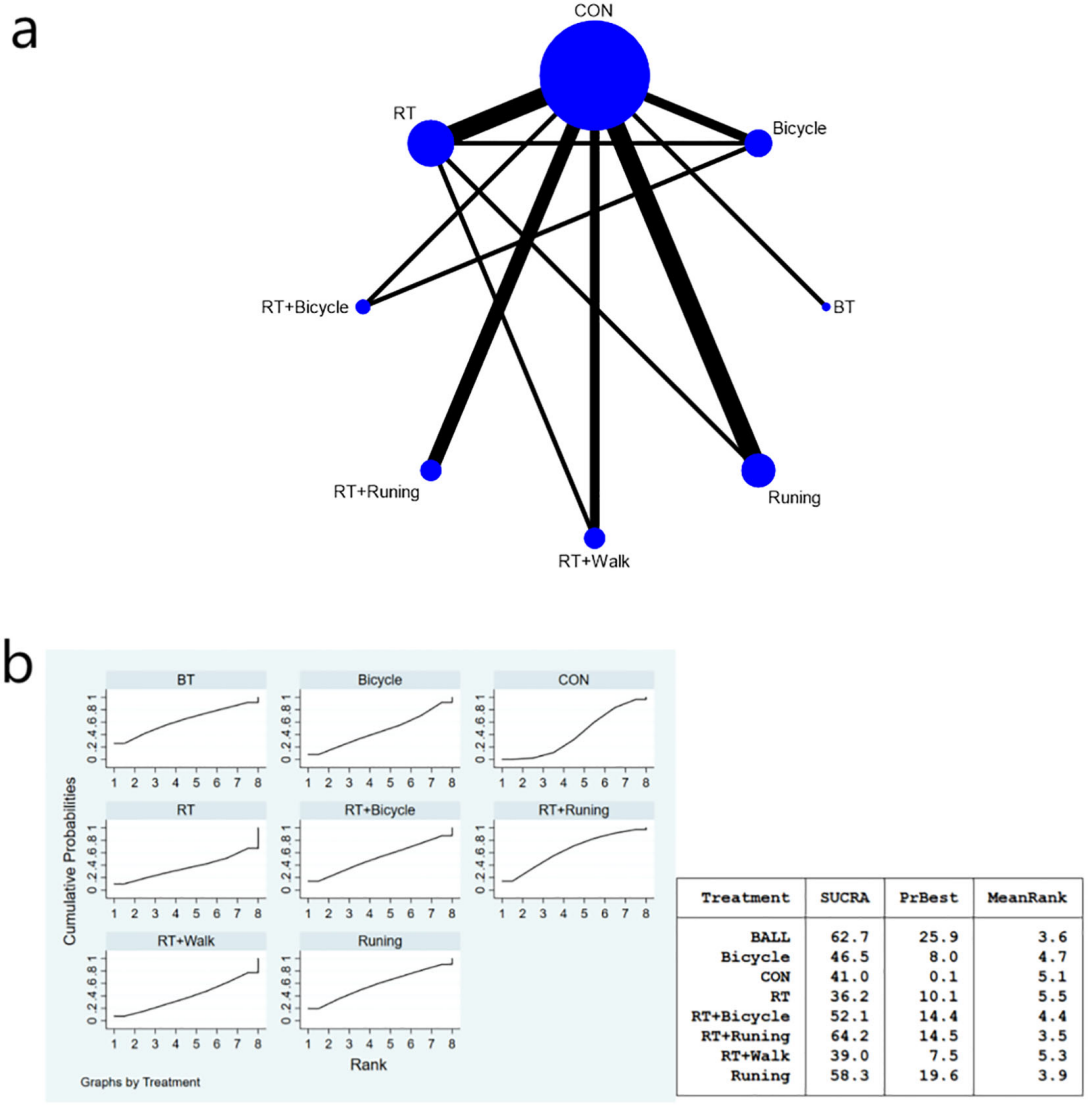
**(a)** Network meta-analysis figure for HOMA-IR; **(b)** SUCRA plot for HOMA-IR.

The complete network diagram is shown in **Figures 3a**, **4a**, and **5a**.

#### 3.4.1 Fasting blood glucose index results of diabetic patients

The meta-analysis results demonstrated that the intervention effect in the bicycle group was superior. Specifically, when comparing the cycling group with both anaerobic and running groups [MD=-46.63, 95%CI (-91.96,-1.29)], and when comparing the running group with the cycling group [MD=-52.19, 95%CI (-101.70,-2.68)], significant differences were observed in favor of the bicycle group’s intervention effect (**Figure 3b**). Furthermore, compared to the control group [MD=-52.64, 95%CI (-95.72,-9.55)], ball games [MD=-56.11, 95%CI (-103.29,-8.94)], and Tai Chi group [MD=-73.02, 95%CI (-120.-17,-25-86)], fasting insulin sensitivity exhibited a more pronounced improvement in insulin sensitivity (**Figure 3b**). Regarding the SUCRA ranking score (**Figure 3b**), cycling practice ranked first with a SUCRA score of 90%. Pairwise comparisons between the interventions are presented in **Table 3**.

**Table 3:** League table on FBG.

Bicycle	RT	RT+Bicycle	RT_Running	Running	CON	RT+Walk	BT	Taichi
Bicycle	-0.96 (-25.93,24.02)	26.37 (-21.52,74.26)	46.63 (1.29,91.96)	52.19 (2.68,101.70)	52.64 (9.55,95.72)	35.71 (-15.40,86.82)	56.11 (8.94,103.29)	73.02 (25.86,120.17)
0.96 (-24.02,25.93)	RT	27.33 (-26.68,81.33)	47.58 (-4.15,99.31)	53.15 (-2.28,108.57)	53.59 (3.82,103.36)	36.67 (-20.19,93.53)	-52.39 (-105.07,0.29)	73.97 (20.64,127.30)
-26.37 (-74.26,21.52)	-27.33 (-81.33,26.68)	RT+Bicycle	20.26 (-36.01,76.52)	25.82 (-33.86,85.50)	26.27 (-28.20,80.74)	9.34 (-51.68,70.36)	-50.85 (-108.21,6.52)	46.65 (-11.10,104.39)
**-46.63 (-91.96,-1.29)**	-47.58 (-99.31,4.15)	-20.26 (-76.52,36.01)	RT+Running	5.56 (-22.62,33.75)	6.01 (-8.11,20.13)	-10.92 (-41.83,20.00)	-53.42 (-108.84,2.01)	26.39 (2.55,50.23)
**-52.19 (-101.70,-2.68)**	-53.15 (-108.57,2.28)	-25.82 (-85.50,33.86)	-5.56 (-33.75,22.62)	Running	0.45 (-23.95,24.84)	-16.48 (-53.24,20.28)	29.75 (-28.03,87.52)	20.83 (-10.22,51.87)
**-52.64 (-95.72,-9.55)**	**-53.59 (-103.36,-3.82)**	-26.27 (-80.74,28.20)	-6.01 (-20.13,8.11)	-0.45 (-24.84,23.95)	CON	-16.93 (-44.43,10.57)	3.48 (-15.90,22.86)	20.38 (1.18,39.57)
-35.71 (-86.82,15.40)	-36.67 (-93.53,20.19)	-9.34 (-70.36,51.68)	10.92 (-20.00,41.83)	16.48 (-20.28,53.24)	16.93 (-10.57,44.43)	RT+Walk	57.07 (3.72,110.42)	37.31 (3.77,70.84)
**-56.11 (-103.29,-8.94)**	52.39 (-0.29,105.07)	50.85 (-6.52,108.21)	53.42 (-2.01,108.84)	-29.75 (-87.52,28.03)	-3.48 (-22.86,15.90)	**-57.07 (-110.42,-3.72)**	BT	-9.49 (-33.45,14.47)
**-73.02 (-120.17,-25.86)**	**-73.97 (-127.30,-20.64)**	-46.65 (-104.39,11.10)	**-26.39 (-50.23,-2.55)**	-20.83 (-51.87,10.22)	**-20.38 (-39.57,-1.18)**	**-37.31 (-70.84,-3.77)**	9.49 (-14.47,33.45)	Taichi

Bold values: indicate statistically significant differences (P < 0.05).

Notably, cycling interventions had longer session durations (mean 90 min) compared to running (mean 45 min) and resistance training (mean 50 min). However, after adjusting for MET-minutes, cycling remained superior in reducing FBG [MD = -38.72, 95%CI (-75.15, -2.29)].

#### 3.4.2 Fasting insulin index results of diabetic patients

In comparison to ball games, resistance exercise demonstrated a significant impact on enhancing insulin sensitivity [MD=-26.71, 95% CI (-51.23, -2.19)].The Qigong exercise group exhibited significant differences compared to the aerobic exercise group [MD=33.04, 95% CI (4.82, 61.26)], bicycle exercise group [MD=30.54, 95% CI (4.41, 56.67)], aerobic walking combined exercise group [MD=26.03, 95% CI (0.40, 51.66)], aerobic running combined exercise group [MD=29.74, 95% CI (4.16, 55.32)], running exercise group [MD=29.68,95%CI(4.10,55.26)], the general control group[MD=28.54,95%CI(5.22,51.86)], and the aerobic and bicycle combined exercise group [MD=28. 21,95%CI(2.30,54.12)], the results suggest that the Qigong exercise intervention had limited impact on improving insulin sensitivity parameters. The bicycle group (SUCRA: 90.7%) exhibited superior efficacy in enhancing insulin sensitivity parameters, as demonstrated in **Figure 4b** of the SUCRA analysis. The effect size of the key comparison indicates that, Resistance training *vs*. Ball training: MD = -26.71 μU/ml, 95%CI (-51.23, -2.19);Qigong *vs*. Control: MD = -28.54 μU/ml, 95%CI (-51.86, -5.22);Cycling *vs*. Control: MD = -2.00 μU/ml, 95%CI (-13.79, 9.79). The MD for Resistance Training versus Ball Training and Qigong versus control was statistically significant. A comparison of the various interventions is shown in **Table 4**.

**Table 4:** League table on fasting insulin.

RT	Bicycle	RT+Walk	RT+Running	Running	CON	RT+Bicycle	BT	QiGong
RT	-2.50 (-13.15,8.15)	-7.01 (-26.12,12.11)	-3.30 (-22.34,15.75)	-3.36 (-22.41,15.70)	-4.50 (-20.38,11.39)	-4.83 (-24.32,14.66)	26.71 (2.19,51.23)	**-33.04 (-61.26,-4.82)**
2.50 (-8.15,13.15)	Bicycle	-4.51 (-20.38,11.37)	-0.80 (-16.59,14.99)	-0.86 (-16.66,14.95)	-2.00 (-13.79,9.79)	-2.33 (-18.65,13.99)	3.01 (-13.10,19.12)	**-30.54 (-56.67,-4.41)**
7.01 (-12.11,26.12)	4.51 (-11.37,20.38)	RT+Walk	3.71 (-11.23,18.65)	3.65 (-11.31,18.61)	2.51 (-8.12,13.14)	2.18 (-13.32,17.68)	6.01 (-13.16,25.17)	**-26.03 (-51.66,-0.40)**
3.30 (-15.75,22.34)	0.80 (-14.99,16.59)	-3.71 (-18.65,11.23)	RT+Running	-0.06 (-10.56,10.44)	-1.20 (-11.70,9.30)	-1.53 (-16.95,13.89)	3.82 (-15.23,22.87)	**-29.74 (-55.32,-4.16)**
3.36 (-15.70,22.41)	0.86 (-14.95,16.66)	-3.65 (-18.61,11.31)	0.06 (-10.44,10.56)	Running	-1.14 (-11.66,9.38)	-1.47 (-16.90,13.96)	7.04 (-12.02,26.09)	**-29.68 (-55.26,-4.10)**
4.50 (-11.39,20.38)	2.00 (-9.79,13.79)	-2.51 (-13.14,8.12)	1.20 (-9.30,11.70)	1.14 (-9.38,11.66)	CON	-0.33 (-11.62,10.96)	-3.83 (-17.84,10.19)	**-28.54 (-51.86,-5.22)**
4.83 (-14.66,24.32)	2.33 (-13.99,18.65)	-2.18 (-17.68,13.32)	1.53 (-13.89,16.95)	1.47 (-13.96,16.90)	0.33 (-10.96,11.62)	RT+Bicycle	7.53 (-24.40,39.45)	**-28.21 (-54.12,-2.30)**
**-26.71 (-51.23,-2.19)**	-3.01 (-19.12,13.10)	-6.01 (-25.17,13.16)	-3.82 (-22.87,15.23)	-7.04 (-26.09,12.02)	3.83 (-10.19,17.84)	-7.53 (-39.45,24.40)	BT	1.83 (-5.75,9.41)
**33.04 (4.82,61.26)**	**30.54 (4.41,56.67)**	**26.03 (0.40,51.66)**	**29.74 (4.16,55.32)**	**29.68 (4.10,55.26)**	**28.54 (5.22,51.86)**	**28.21 (2.30,54.12)**	-1.83 (-9.41,5.75)	QiGong

Bold values: indicate statistically significant differences (P < 0.05).

#### 3.4.3 Results of the HOMA-IR index for diabetic patients

The meta-analysis chart results (**Figure 5b**) revealed no statistically significant differences in the reduction of HOMA-IR index among the intervention groups. The SUCRA value indicated that the combination of aerobic exercise and running exhibited the highest ranking for reducing HOMA-IR values, thus proving to be the most effective approach compared to other exercises (SUCRA: 64.2%). Ball games ranked next (SUCRA: 62.7%), as shown in **Figure 4b**. HOMA-IR changes versus control:RT+Running: MD = -1.20, 95%CI (-11.70, 9.30); ball training: MD = -3.83, 95%CI (-17.84, 10.19); Cycling: MD = -0.10, 95%CI (-3.93, 3.73); Although RT+Running had the highest SUCRA ranking, its effect versus control did not reach statistical significance (95%CI crosses zero). The pairwise comparison of the interventions is presented in **Table 5**.

**Table 5:** League table on HOMA-IR.

RT+Running	BT	Running	RT+Bicycle	Bicycle	CON	RT+Walk	RT
RT+Running	0.96 (-5.50,7.42)	0.20 (-4.10,4.49)	0.51 (-3.79,4.80)	0.88 (-3.58,5.33)	0.98 (-1.30,3.25)	0.19 (-4.13,4.50)	1.58 (-4.20,7.35)
-0.96 (-7.42,5.50)	BT	1.70 (-4.87,8.27)	-1.27 (-7.31,4.77)	-0.17 (-5.47,5.13)	1.00 (-4.45,6.45)	-1.38 (-7.82,5.06)	1.10 (-2.78,4.98)
-0.20 (-4.49,4.10)	-1.70 (-8.27,4.87)	Running	0.31 (-4.85,5.47)	0.68 (-4.61,5.97)	0.78 (-2.87,4.43)	-0.01 (-5.18,5.16)	1.38 (-5.06,7.82)
-0.51 (-4.80,3.79)	1.27 (-4.77,7.31)	-0.31 (-5.47,4.85)	RT+Bicycle	0.37 (-4.92,5.66)	0.47 (-3.18,4.12)	-0.32 (-5.49,4.85)	1.07 (-5.37,7.51)
-0.88 (-5.33,3.58)	0.17 (-5.13,5.47)	-0.68 (-5.97,4.61)	-0.37 (-5.66,4.92)	Bicycle	0.10 (-3.73,3.93)	-0.69 (-5.99,4.61)	0.70 (-2.97,4.37)
-0.98 (-3.25,1.30)	-1.00 (-6.45,4.45)	-0.78 (-4.43,2.87)	-0.47 (-4.12,3.18)	-0.10 (-3.93,3.73)	CON	-0.79 (-4.46,2.88)	0.60 (-4.70,5.90)
-0.19 (-4.50,4.13)	1.38 (-5.06,7.82)	0.01 (-5.16,5.18)	0.32 (-4.85,5.49)	0.69 (-4.61,5.99)	0.79 (-2.88,4.46)	RT+Walk	1.39 (-5.06,7.84)
-1.58 (-7.35,4.20)	-1.10 (-4.98,2.78)	-1.38 (-7.82,5.06)	-1.07 (-7.51,5.37)	-0.70 (-4.37,2.97)	-0.60 (-5.90,4.70)	-1.39 (-7.84,5.06)	RT

Bicycle, bicycle training; RT+Running, resistance training and Running; BT, ball training; RT, resistance training; RT+Running, resistance training and Running; RT+bicycle, resistance training and bicycle; RT+walk, CON, resistance training and walk; control group (no exercise).

### 3.5 Publication bias test

**Figure 6:**
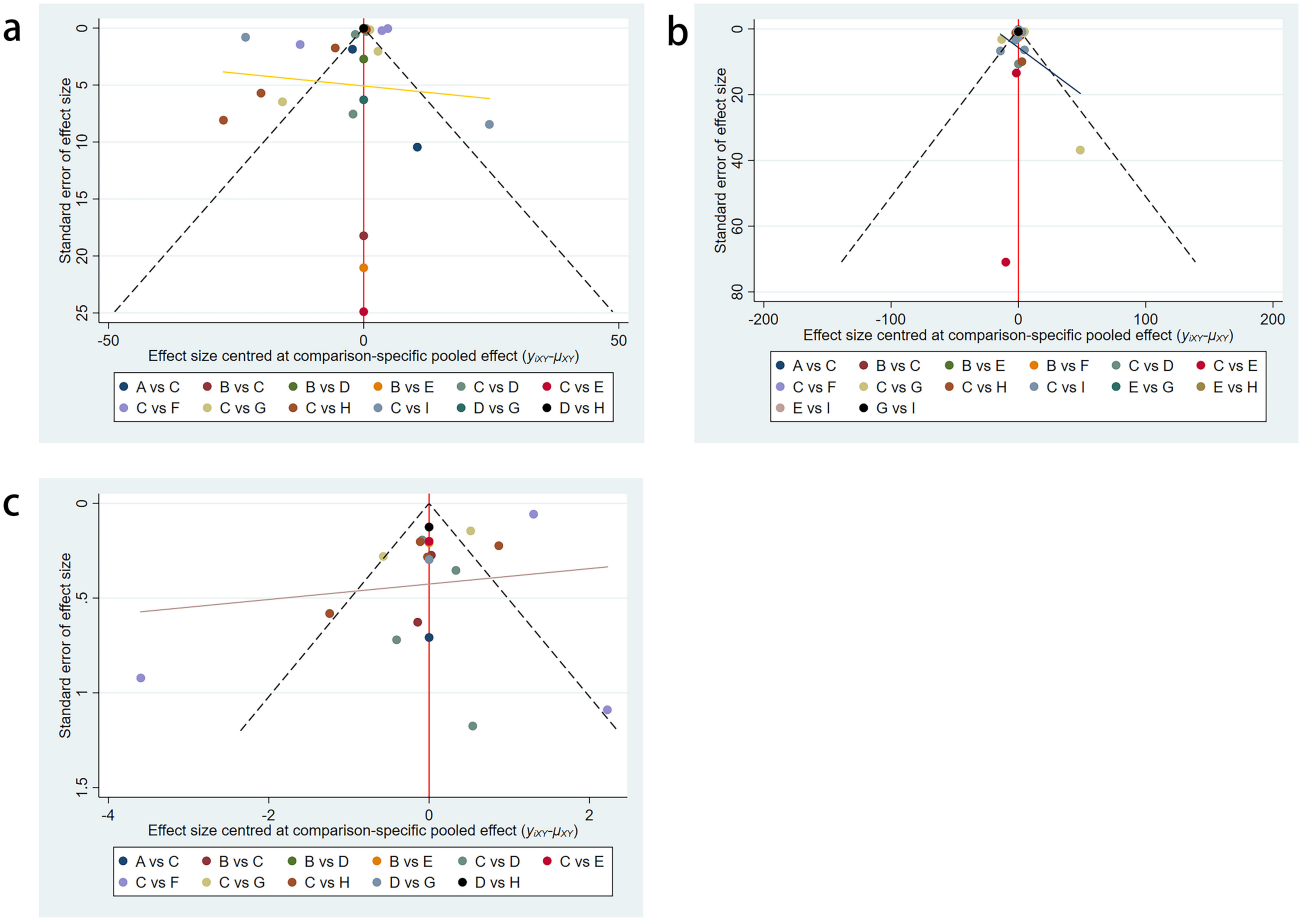
Funnel plot on publication bias. **(a)** FBG; **(b)** fasting insulin; **(c)** HOMA-IR.

The included trials were assessed using the Cochrane risk assessment tool and were determined to have a low-to-moderate risk of bias. Additionally, no significant publication bias was observed in the funnel plots (**Figures 6a–c**).

## 4 Discussion

In this meta-review and meta-analysis, we incorporated data from studies conducted across multiple continents, including the United States, Europe, Asia, and Australia, to augment the sample size and enhance the generalizability of our findings. By comparing the effects of nine different exercise interventions, we observed that cycling, resistance exercise, and combined resistance with running exercise exhibited comparatively superior enhancements. Specifically, cycling showed the largest FBG reduction [MD = -52.64 mmol/L *vs*. control], resistance training significantly improved insulin sensitivity over ball games [MD = -26.71 μU/ml], and RT+Running had the highest probability (SUCRA=64.2%) for HOMA-IR reduction despite non-significant effects versus control [MD = -1.20].

By comparing the effects of nine different exercise interventions on fasting blood glucose, fasting insulin, and HOMA-IR levels among patients with diabetes, we observed that cycling, resistance exercise, and combined resistance with running exercise demonstrated relatively superior improvements in glycemic control indicators, including FPG, FI, and HOMA-IR index. Cycling is most likely to reduce fasting plasma glucose (FPG) levels, which is consistent with previous evidence indicating that cycling recruits a more significant number of type I muscle fibers and improves glucose utilization (37, 38). The extensive engagement of muscles and the absence of weight-bearing characteristics make cycling a safer and more effective exercise option for individuals with type 2 diabetes mellitus (T2DMM) (39). Utilizing bicycles mobilizes large muscle groups and eliminates leg weight-bearing and ground friction, making it remarkably safe and effective for patients with T2DMM. Cycling elicits greater recruitment of type I muscle fibers, which demonstrate higher insulin sensitivity and GLUT4 density (4, 39). Li et al. demonstrated that both high-intensity interval cycling and moderate-intensity cycling significantly reduced fasting glucose in T2DMM patients (40).

During exercise under normoglycemic-hyperinsulinemic conditions, skeletal muscles account for nearly all human glucose uptake. The increase in muscle glucose uptake during exercise is attributed to enhanced contraction activity and increased blood flow within the muscles, which facilitates glucose transport (41). The higher level of glucose utilization observed during cycling compared with running may be due to the greater contraction activity resulting from the larger active muscle mass. Muscle fiber recruitment and glycogen utilization patterns differ among various forms of exercise. It has been discovered that the effect on muscle glycogen supply by type I fibers was superior in the group undergoing cycling interventions compared to those undergoing running interventions. Type I fibers possess higher insulin content, are more sensitive to insulin stimulation, and can recruit more GLUT4 transport proteins, thereby enhancing the skeletal muscle’s ability to take up and transport glucose, an effect associated with increased insulin-stimulated glucose uptake capability (37, 39). These findings suggest that cycling elicits greater recruitment of type I fibers and higher glucose utilization than running does.

This study suggests that resistance training is beneficial for improving insulin utilization in patients with type 2 diabetes. Compared to conventional exercise, resistance training can more effectively promote skeletal muscle glucose utilization and uptake due to its ability to increase muscle mass and cross-sectional area (42, 43), thereby facilitating insulin signaling and peripheral tissue glucose uptake (44, 45). Resistance training can augment glucose phosphorylation in skeletal muscle cells, facilitating the conversion of blood sugar into simple sugars, thereby promoting optimal insulin secretion and maintaining blood sugar homeostasis (44, 45). Long-term (>12 weeks) high-intensity resistance training has been shown to significantly enhance insulin sensitivity and sustain physical function for a duration that surpasses that of aerobic exercise (46). The findings of various studies have demonstrated that engagement in resistance exercise can significantly enhance metabolic health during weight recovery, including the reduction of fasting blood glucose levels and enhancement of insulin sensitivity (47). In a 24-week study, a comparison between resistance training and aerobic exercise revealed that the former enhanced insulin sensitivity and glucose uptake in muscles mediated by insulin (46). In general, resistance training enhances insulin sensitivity and improves fasting glucose levels in individuals diagnosed with type 2 diabetes (46, 48).

The combination of running and anaerobic exercise demonstrated superior efficacy in alleviating insulin resistance, as supported by a significant reduction in the HOMA-IR index, indicating an enhanced improvement in insulin sensitivity. The underlying mechanisms potentially involve augmented lipid oxidation and glycogen utilization (38), improved mitochondrial function (49), and enhanced muscle mass and cardiorespiratory fitness (40). Type 2 diabetes is characterized by insulin resistance (IR) and relative insulin insufficiency, leading to glucose intolerance and subsequent elevation of blood glucose levels (40). However, preserving islet β-cell function may be pivotal in preventing T2DM onset (49, 50). Notably, the combined impact of running and resistance training on β-cell function surpasses that achieved through either aerobic or resistance training alone (51), likely attributable to the prolonged duration and heightened intensity associated with combined training regimens. Low-load high-repetition resistance training has emerged as an alternative form of aerobic-based resistance training capable of promoting muscle hypertrophy and strength gains similar to those observed with high-load low-repetition protocols (51, 52). In the context of combined training approaches, increased fat loss during resistance exercise aids in augmenting glucose uptake while concurrently enhancing skeletal muscle mitochondrial oxidative capacity. This synergistic effect maximizes reductionions in body fat content while expediting glycogen consumption during aerobic exercise sessions (53).

## 5 Advantages and limitations

The methodology employed in this study was highly rigorous and systematic. We conducted a comprehensive search across five electronic databases, strictly adhering to predefined criteria, and identified 21 articles encompassing a substantial sample size of 1140 patients with diabetes. To ensure accuracy, the selected articles underwent double-checking procedures, and we incorporated various specific joint exercise measures targeting both aerobic and anaerobic activities, thereby providing updated and more comprehensive evidence-based recommendations on how exercise can effectively reduce blood glucose levels and enhance insulin sensitivity.

Nevertheless, certain limitations of this meta-analysis should be acknowledged.

1. Cycling duration confounder: The observed superiority of cycling (e.g., FBG MD=-52.64 *vs* control) must be interpreted in the context of its typically longer session durations (35 min-3 h *vs* 30 min-2 h for running). While our MET-adjusted analysis suggested that duration alone did not fully explain efficacy (54), energy expenditure differentials remained a potential confounder.
2. Surrogate markers: Reliance on FBG/FI/HOMA-IR rather than gold-standard measures (for example, hyperinsulinemic-eug clamps);
3. Language bias: The inclusion of the CNKI database may limit generalizability, although funnel plots showed symmetry.
4. Blinding impossibility: Participant blinding was unattainable due to the nature of the exercise intervention.

Future directions: (a) Match interventions by MET-minutes to isolate modality effects; (b) validate findings with direct insulin sensitivity measures; (c) extend follow-up beyond 6 months.

## 6 Conclusion

Our study conducted a systematic review and network meta-analysis to compare the effects of different exercise interventions on glycemic control in patients with diabetes. The results demonstrated that cycling, resistance training, and combined resistance and aerobic training effectively improved fasting blood glucose levels, insulin levels, and insulin resistance. These findings have significant implications for the management of diabetes. We recommend prioritizing cycling to reduce blood glucose levels, incorporating resistance training to enhance insulin sensitivity, and implementing combined training to address insulin resistance. Exercise has been proven effective in regulating glycemia and should be widely recommended as an essential non-pharmacological treatment for individuals with diabetes.

Future studies should further validate the benefits of diverse exercise regimens on blood glucose regulation in a broader population. The genetic background and type of diabetes may influence individual variations in response to exercise interventions; thus, we advocate for future trials with expanded sample sizes encompassing various ethnicities to corroborate our current findings. Additionally, exploring the interaction between exercise and antidiabetic drugs is imperative. Longitudinal trials with larger sample sizes are also necessary to investigate the long-term effects of exercise interventions on maintaining blood glucose control among patients with diabetes while providing more personalized exercise recommendations.

## Data Availability

The original contributions presented in the study are included in the article/supplementary material. Further inquiries can be directed to the corresponding authors.
